# Supplementary material for: Clinically Relevant Chemotherapeutics Have the Ability to Induce Immunogenic Cell Death in Non-Small Cell Lung Cancer
Source: Cells. 2020 Jun 16;9(6):1474. doi: 10.3390/cells9061474 (PMC7349161; doi:10.3390/cells9061474)
Supplement: Supplementary file 1 [file cells-09-01474-s001.zip › Supplementary File_Cells.docx]

**Supplementary materials and methods
*Cytokine secretion***Supernatants were collected from the co-cultures of NSCLC cells and dendritic cells (DCs). TNF-α, IFN-β, IL-6 and TGF-β secretion were measured using electrochemiluminescence detection on a SECTOR3000 (MesoScale Discovery/MSD, Discovery Workbench 4.0 software), as previously described [1]. Standards and samples were measured in duplicate and the assay was performed according to the manufacturer’s instructions.

**Supplementary figures**


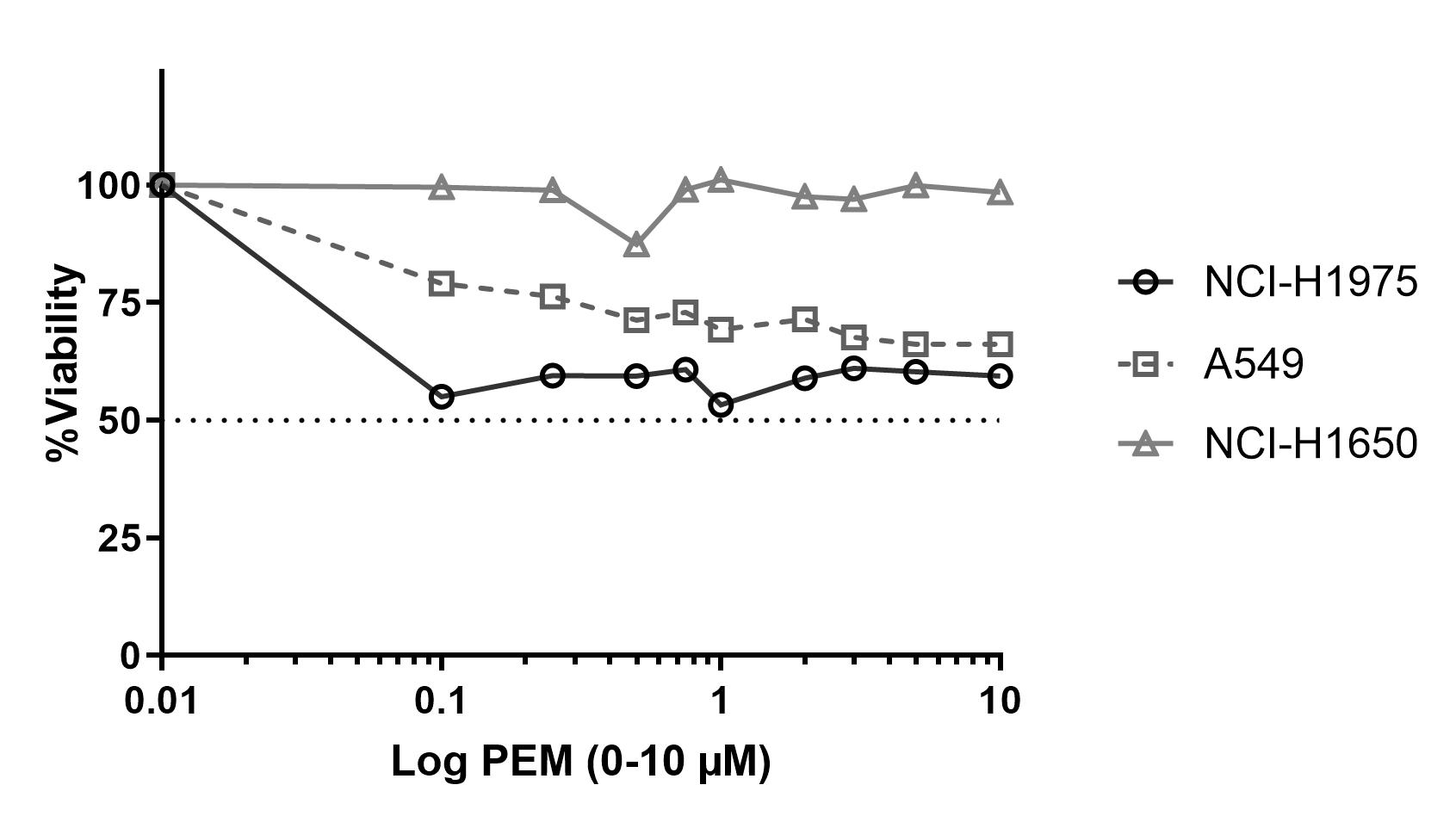


**Figure S1.** Dose-response curves of pemetrexed in human NSCLC cell lines.
Survival curve after 72 h of treatment with pemetrexed (PEM; 0-10 µM) in the NCI-H1975, A549 and NCI-H1650 cell line. The experiment was performed once.


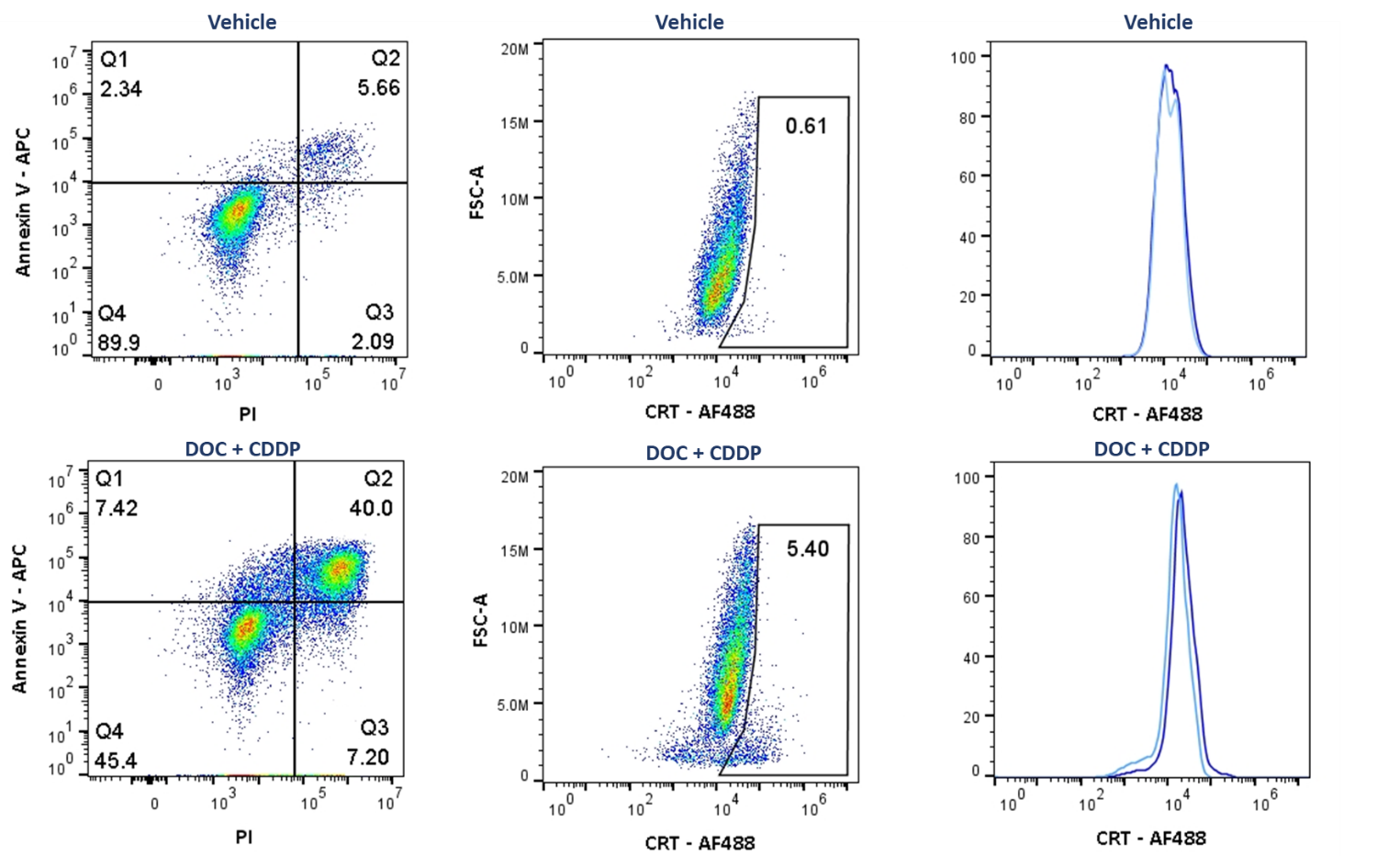


**Figure S2.** Gating strategy of ecto-CALR^+^ cells in NSCLC cell lines.
Two examples of the gating strategy are shown for vehicle (upper plots) and docetaxel + cisplatin (DOC + CDDP) treatment of NCI-H1975 cells (lower plots). Live cells were gated based on propidium iodide (PI)^-^ and Annexin V^+/-^ populations (left plots). Percentages (%) of ecto-calreticulin (CALR)^+^ cells were determined by staining with CALR-AF488 (middle plots). Prior to subtracting % ecto-CALR^+^ cells of vehicle, isotype controls (light blue) were subtracted from each condition (dark blue; right plots).


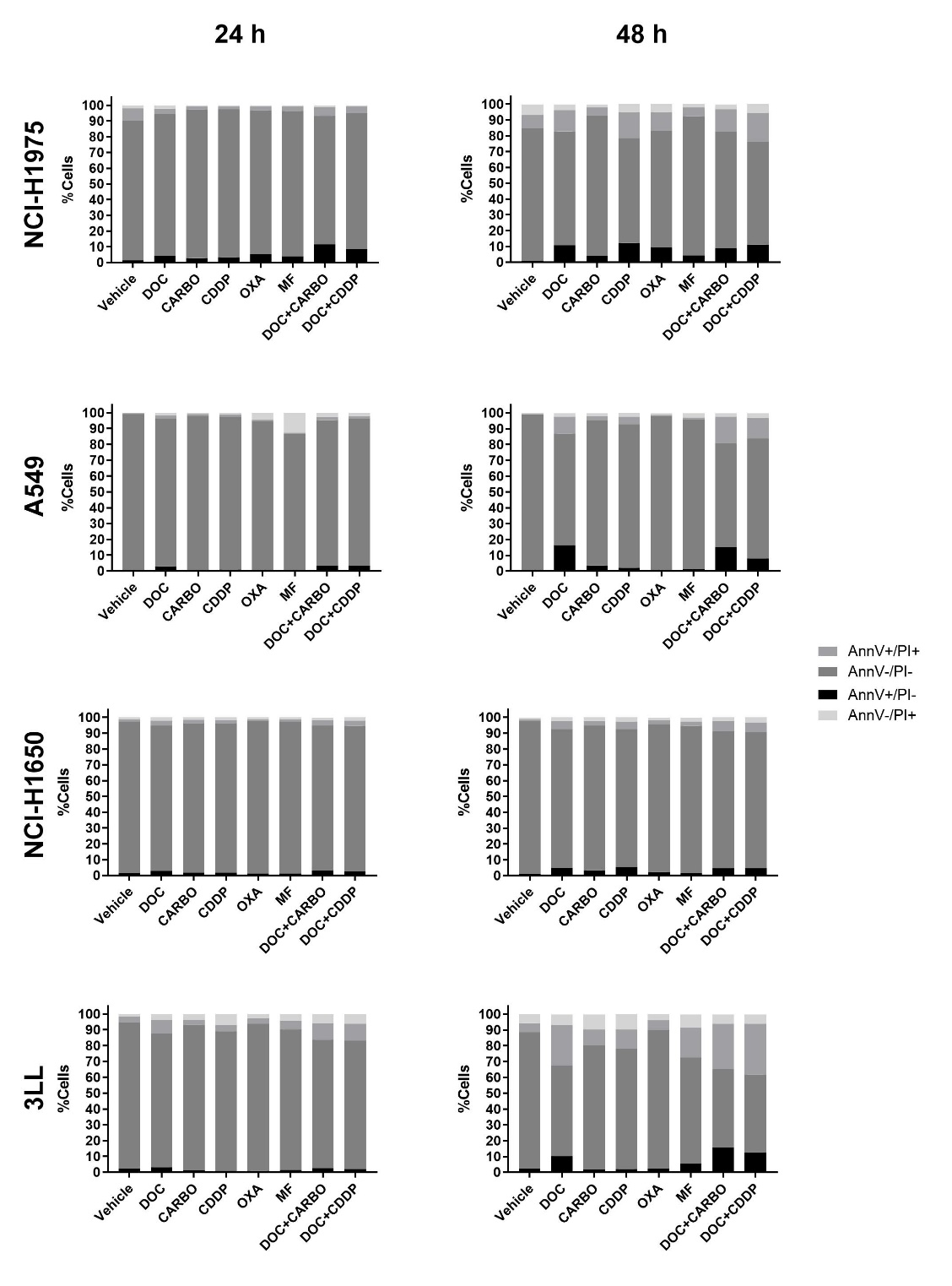


**Figure S3.** NSCLC cell staining with AnnV/PI after 24 h and 48 h treatment with chemotherapy**.**Examples of AnnexinV (AnnV)/PI stainings of NCI-H1975, A549, NCI-H1650 and 3LL cells are shown after 24 h and 48 h of treatment with the IC_50_-72h of docetaxel (DOC), carboplatin (CARBO), cisplatin (CDDP), oxaliplatin (OXA) and mafosfamide (MF) or treatment with the IC50 of DOC and IC_40_-72h value of either CARBO or CDDP.


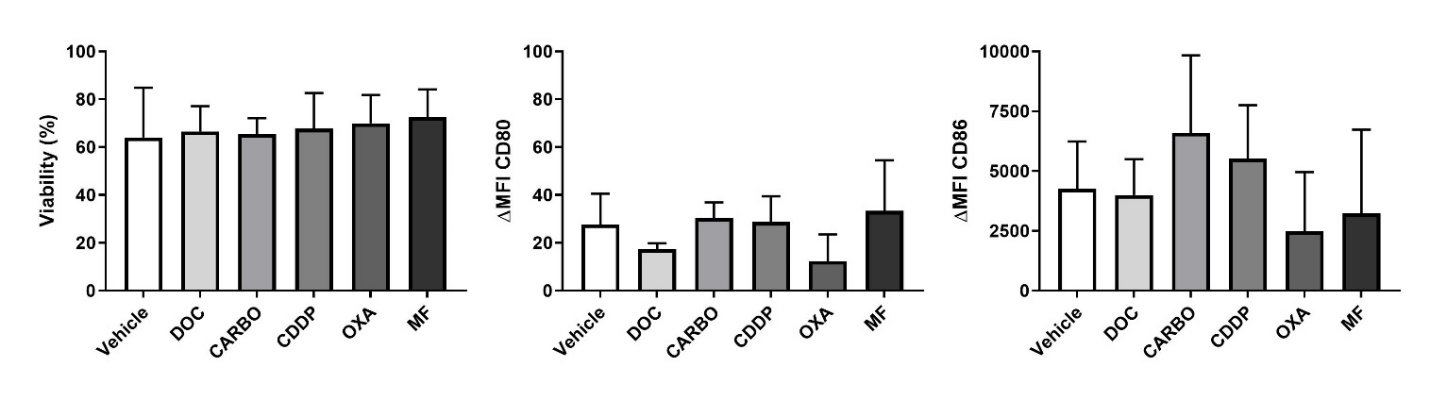


**Figure S4.** DCs viability and maturation status after treatment with chemotherapy.
Viability in percentage (%) and maturation status (CD80, CD86) of dendritic cells (DCs), from three healthy donors, were assessed after 48 h treatment with vehicle (PBS) or the IC_50_-72h of docetaxel (DOC), carboplatin (CARBO), cisplatin (CDDP), oxaliplatin (OXA) and mafosfamide (MF). Data of maturation markers are expressed as delta mean fluorescence intensity (ΔMFI), which was calculated by subtracting the isotype control of each condition. Error bars represent the standard deviation. Experiments were performed in triplicate. No statistically significant differences were present between the different conditions.


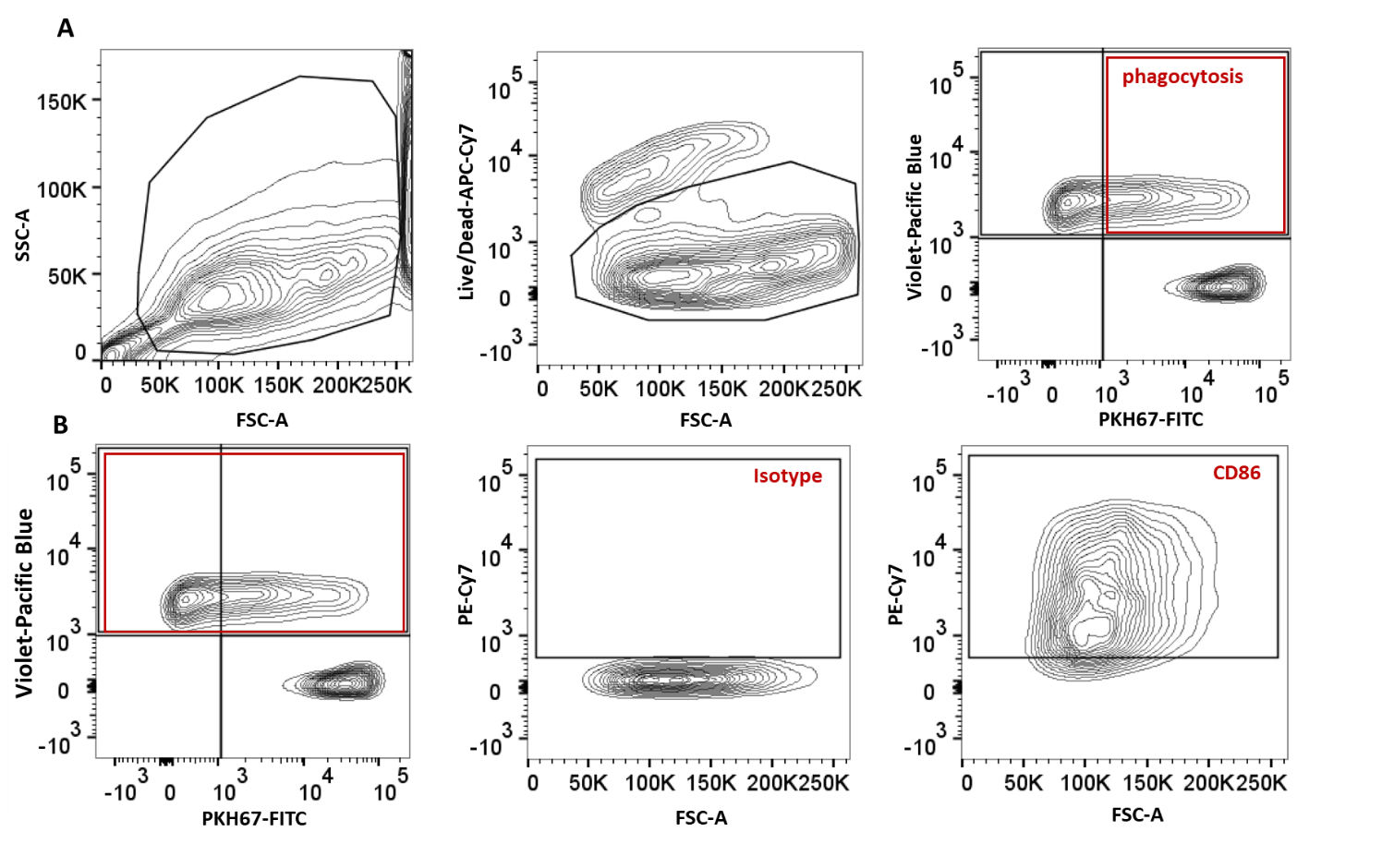


**Figure S5.** Gating strategy of phagocytosis of NSCLC cells and DC maturation.
(a) A gating example is shown of the gating strategy for phagocytosis. PKH67^+^ NSCLC cells taken up by violet-labeled DCs are expressed as the %violet^+^PKH67^+^ cells within the violet^+^ population.
(b) A gating example is shown for expression of the maturation marker CD86. Expression is determined within the violet^+^ population. The gating is determined by comparing to isotype expression.


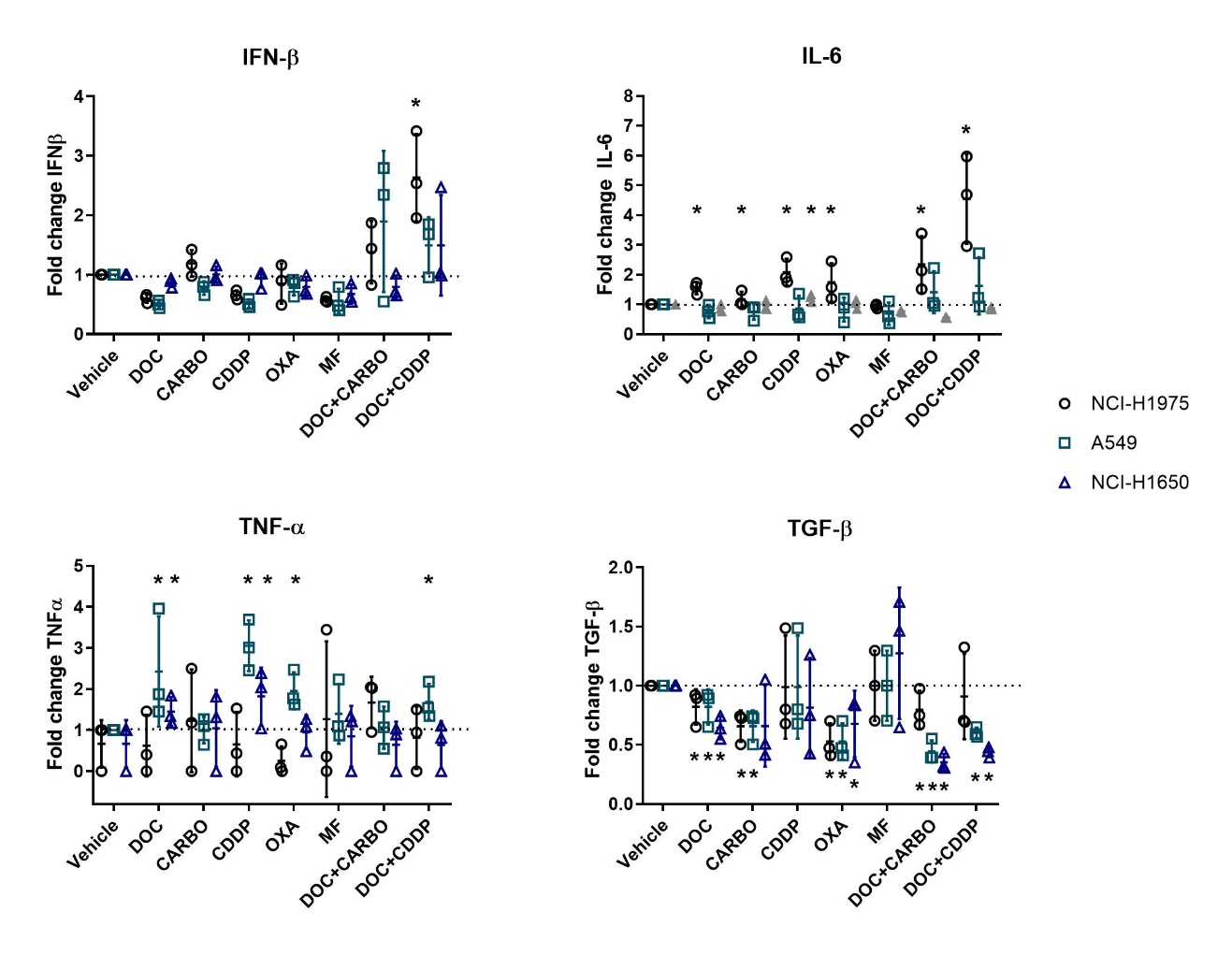


**Figure S6.** Cytokine secretion in supernatant from co-cultures of NSCLC cells and DCs after treatment with chemotherapy.
TGF-β levels (concentration range 300-3000 pg/mL), TNF-α (concentration range 1-16 pg/mL), IFN-β (concentration range 10-150 pg/mL) and IL-6 (concentration range 5-950 pg/mL) were assessed after 72 h of treatment with the IC_50_-72h of docetaxel (DOC), carboplatin (CARBO), cisplatin (CDDP), oxaliplatin (OXA) and mafosfamide (MF) or treatment with the IC_50_-72h of DOC and IC_40_72h value of either CARBO or CDDP in the NCI-H1975, A549 and NCI-H1650 cell lines. Data are represented as fold change. *, For TGF-β: p ≤ 0.05 if decreased compared to vehicle. For IL-6, TNF-α and IFN-β: p ≤ 0.05 if increased compared to vehicle. The error bars represent the standard deviation. Each symbol represents an independent experiment. Grey symbols represent data points above the fit curve.

1. De Waele, J.; Marcq, E.; Van Audenaerde, J.R.; Van Loenhout, J.; Deben, C.; Zwaenepoel, K.; Van de Kelft, E.; Van der Planken, D.; Menovsky, T.; Van den Bergh, J.M.; et al. Poly(I:C) primes primary human glioblastoma cells for an immune response invigorated by PD-L1 blockade. *Oncoimmunology* **2018**, *7*, e1407899.
