# Supplementary figures and images for: Clinically Relevant Chemotherapeutics Have the Ability to Induce Immunogenic Cell Death in Non-Small Cell Lung Cancer
Source: Cells. 2020 Jun 16;9(6):1474. doi: 10.3390/cells9061474 (PMC7349161; doi:10.3390/cells9061474)

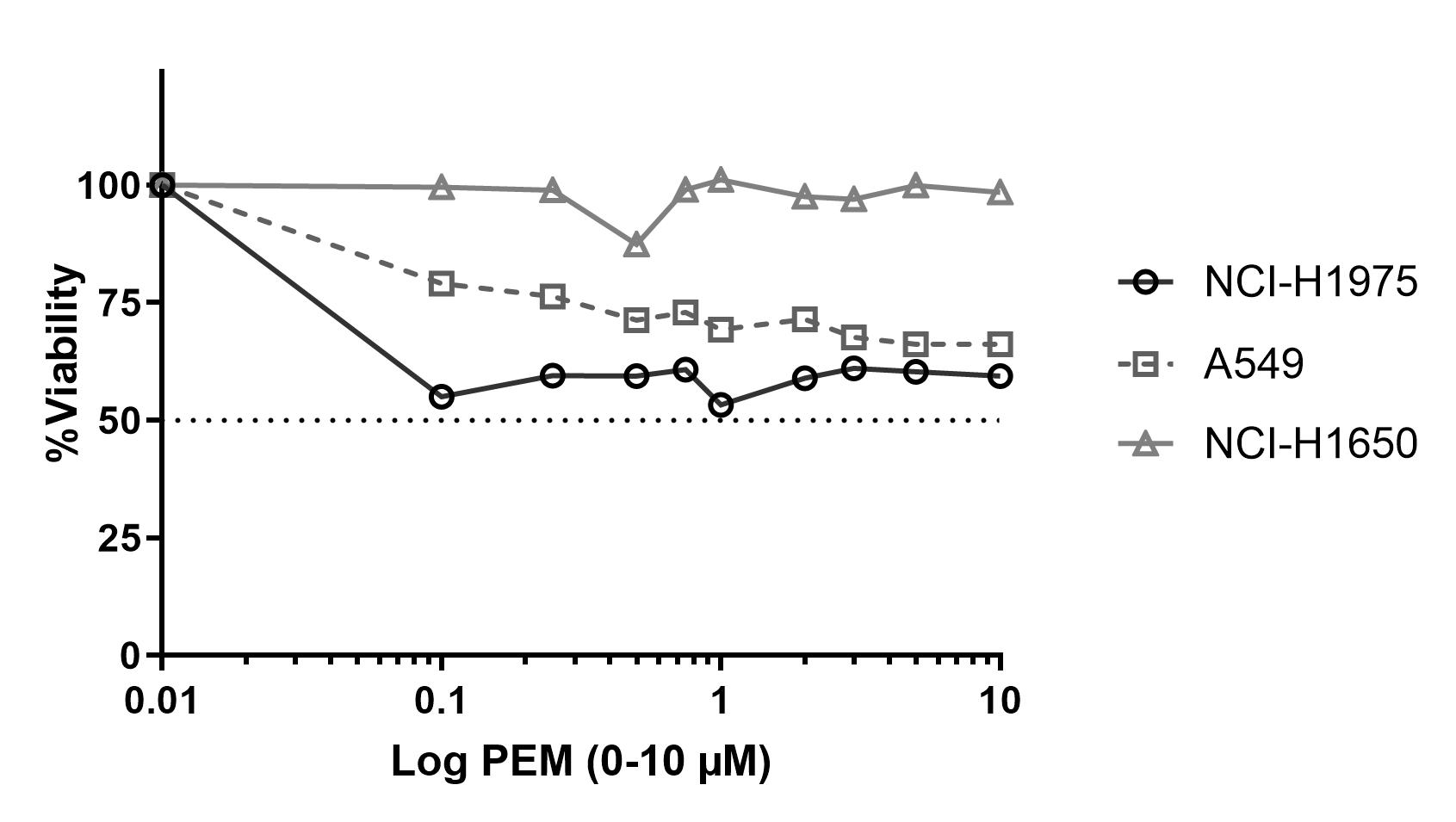

Supplement: Supplementary file 1 [file cells-09-01474-s001.zip › Figure S1.jpg]

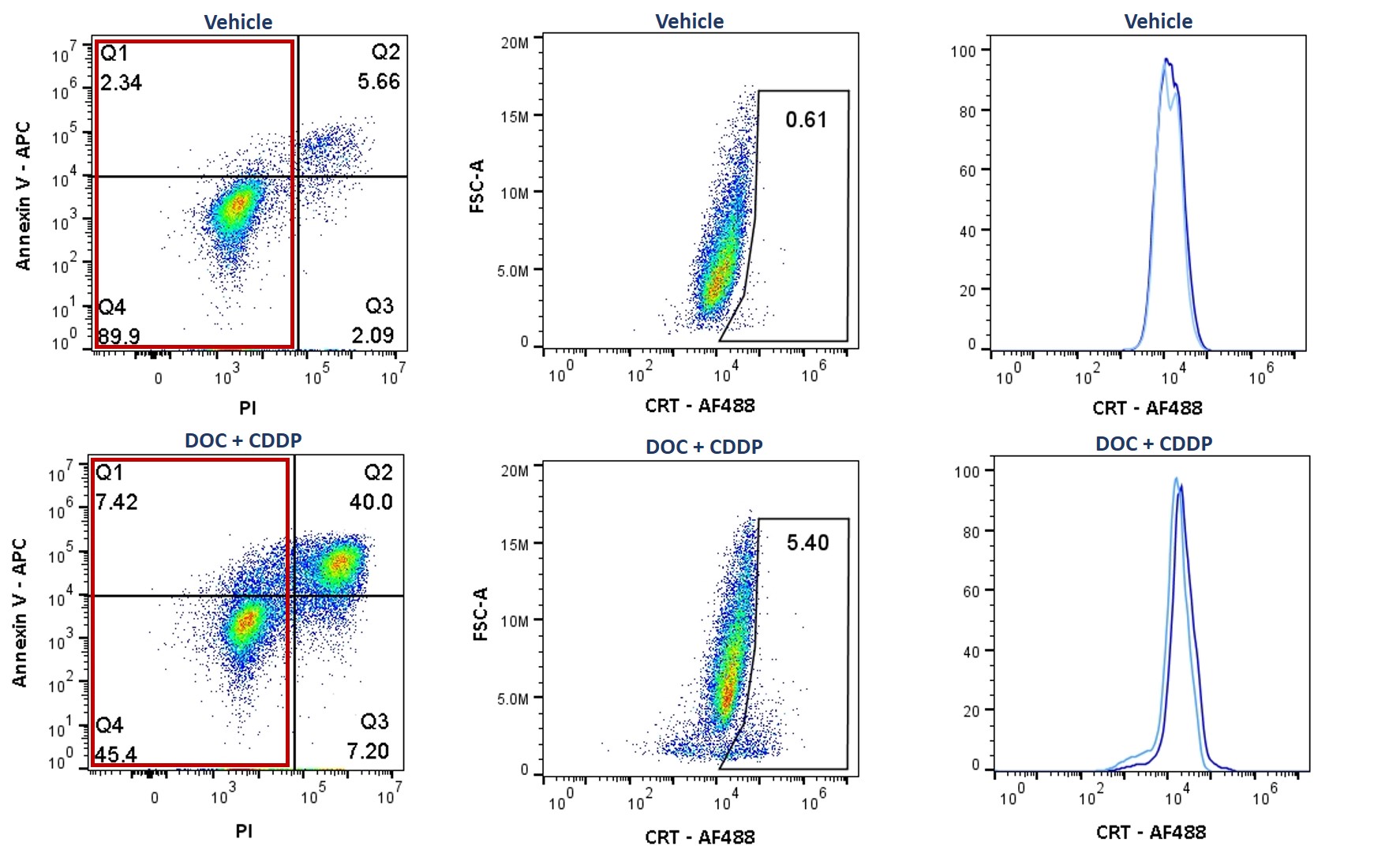

Supplement: Supplementary file 1 [file cells-09-01474-s001.zip › Figure S2.jpg]

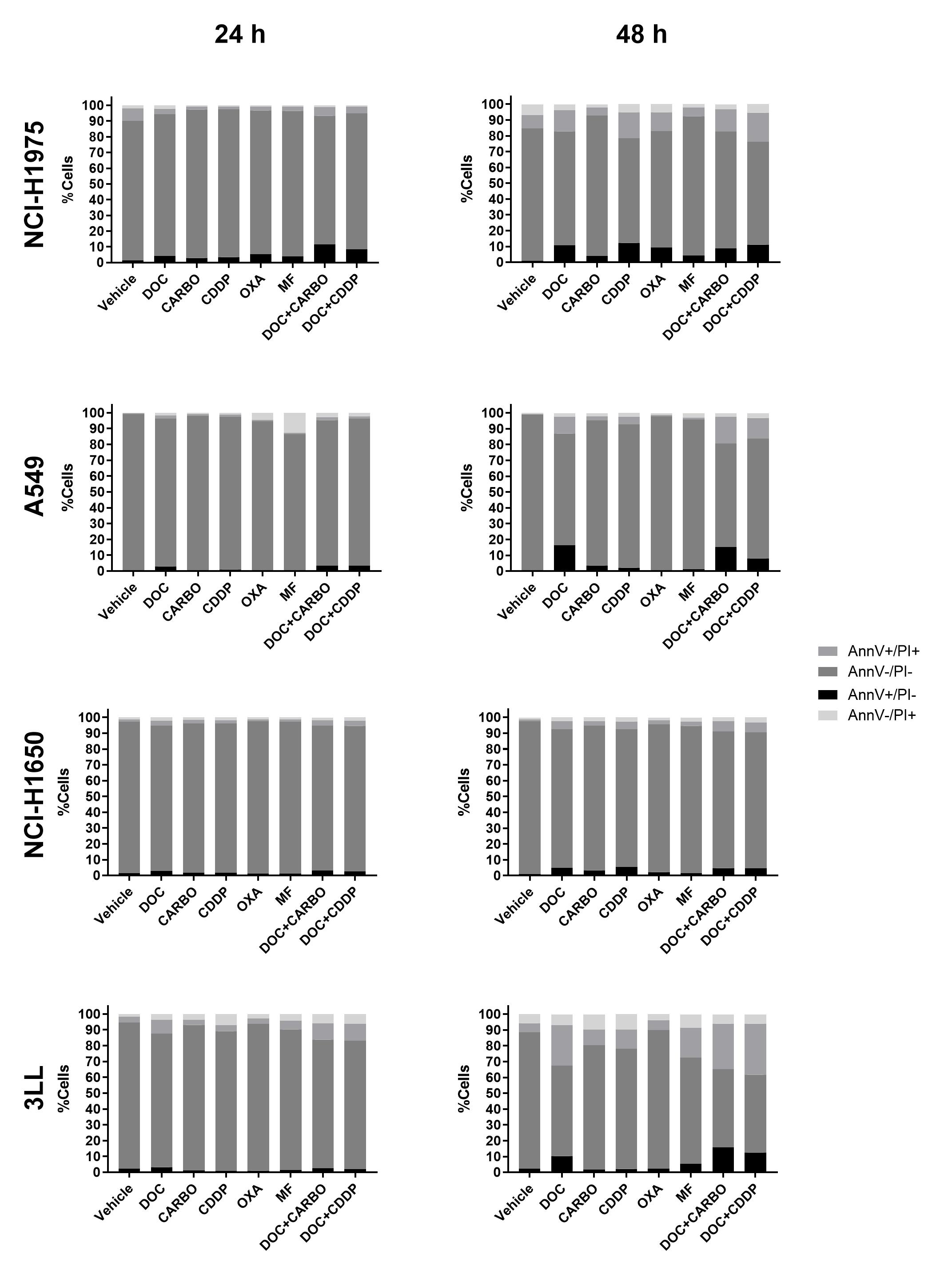

Supplement: Supplementary file 1 [file cells-09-01474-s001.zip › Figure S3.jpg]

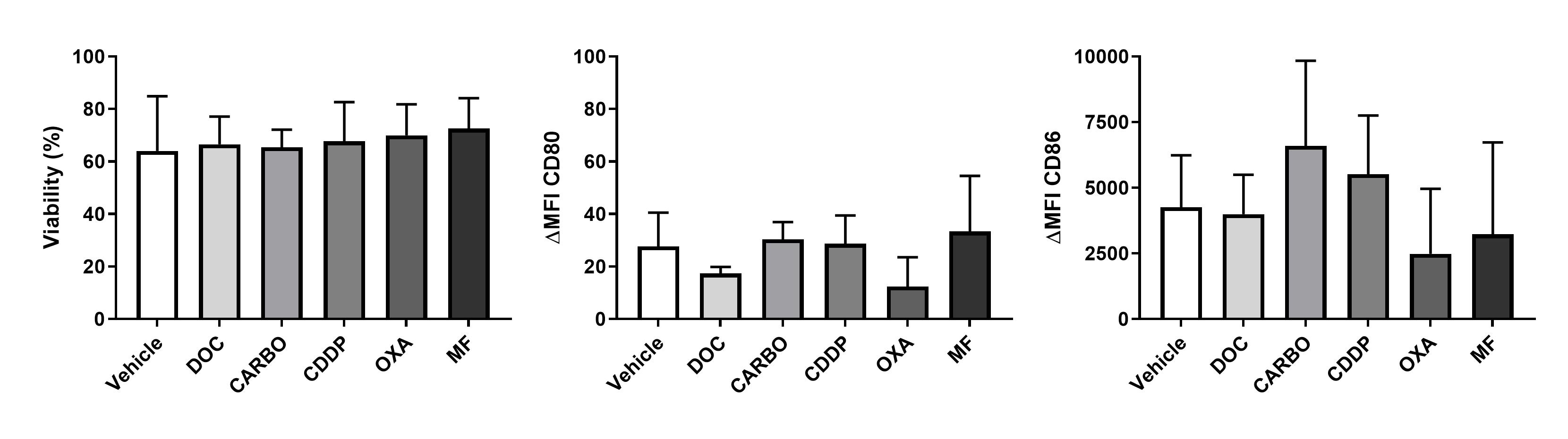

Supplement: Supplementary file 1 [file cells-09-01474-s001.zip › Figure S4.jpg]

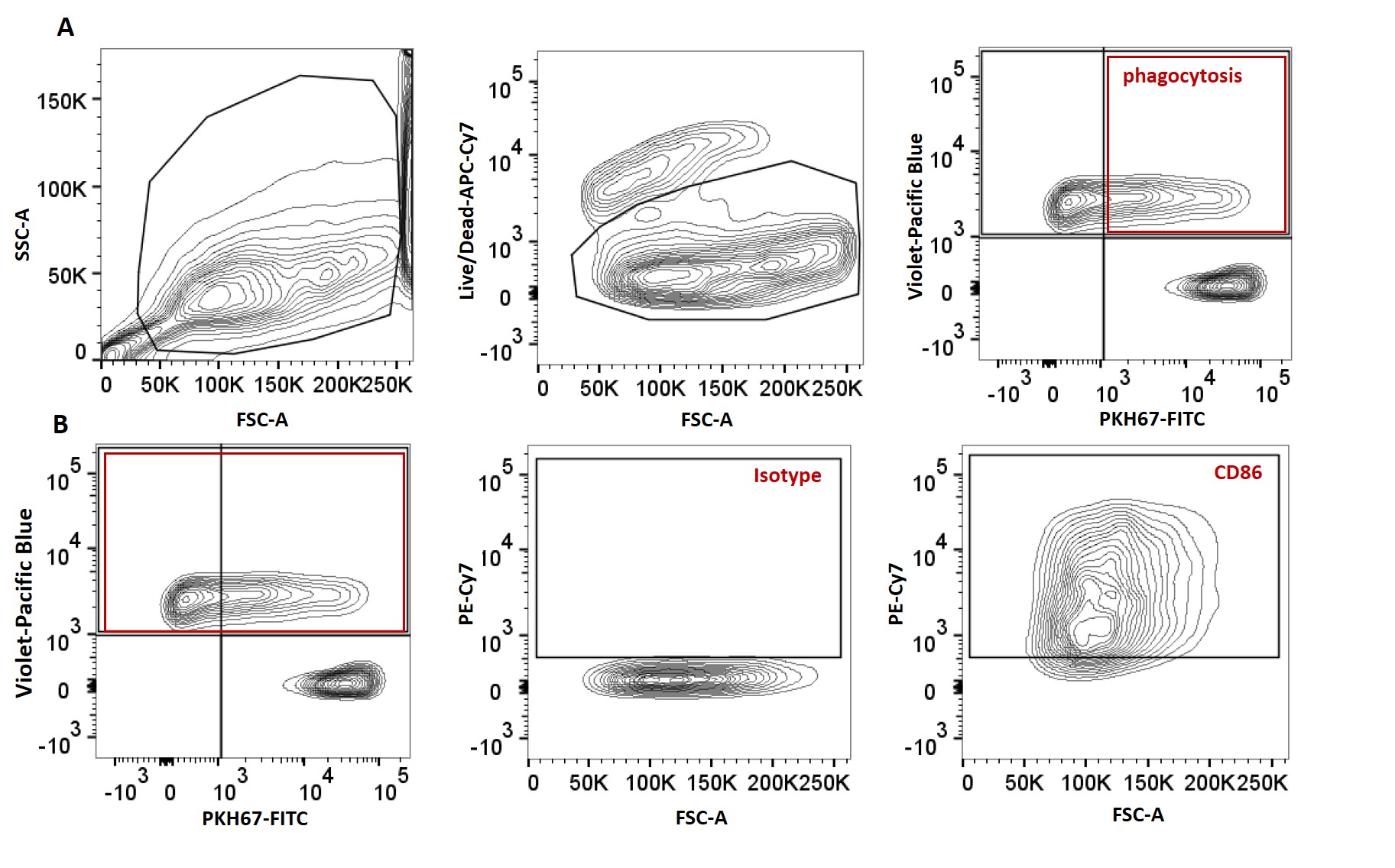

Supplement: Supplementary file 1 [file cells-09-01474-s001.zip › Figure S5.jpg]

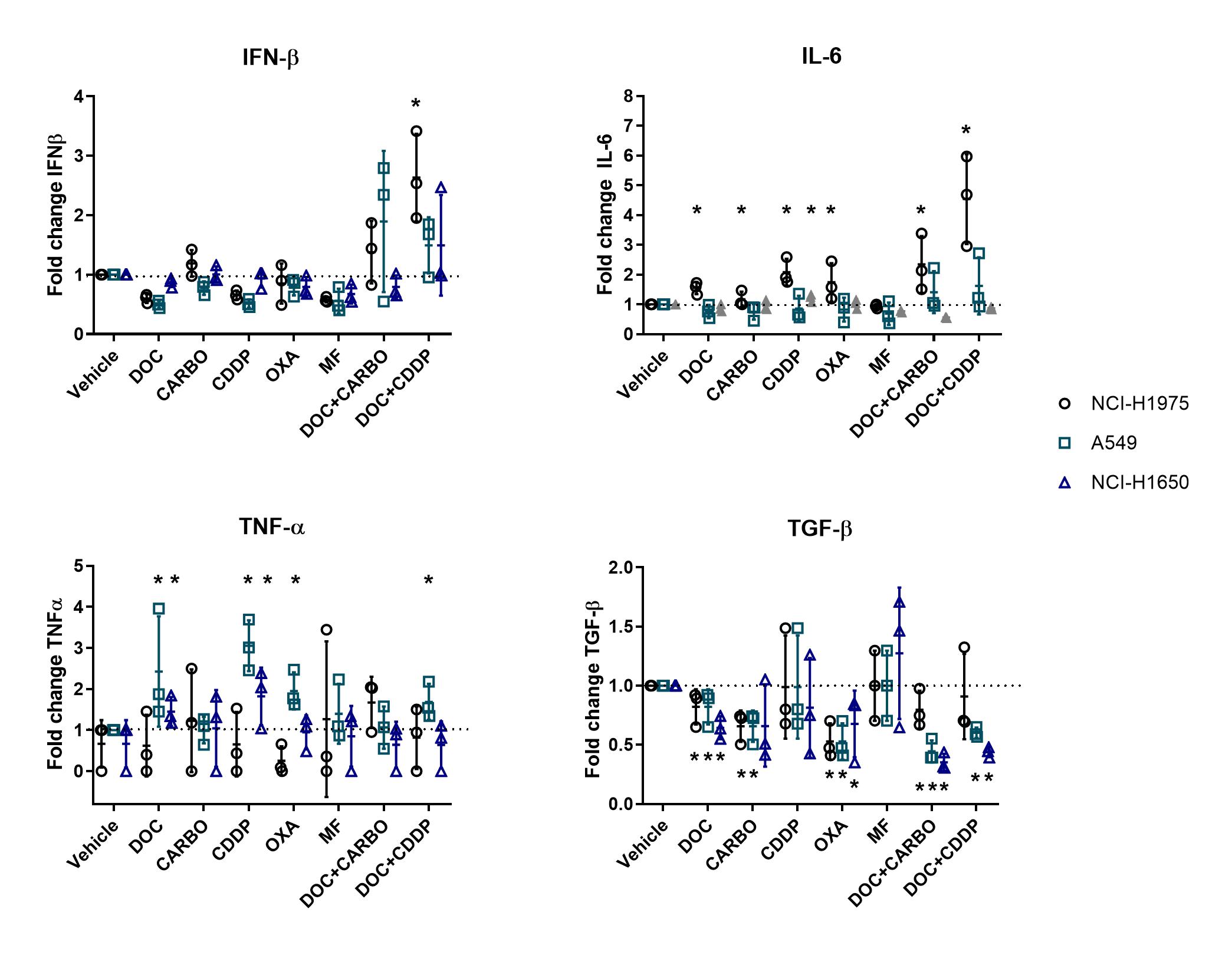

Supplement: Supplementary file 1 [file cells-09-01474-s001.zip › Figure S6.jpg]
